# Laryngomalacia surgery: a series from a tertiary pediatric hospital

**DOI:** 10.5935/1808-8694.20130144

**Published:** 2015-10-08

**VOLUME 78 ISSUE 6 - NOV/DEC 2012**

*José Faibes Lubianca Netto • Renata Loss Drummond • Luciana Pimentel Oppermann • Fernando Stahl Hermes • Rita Carolina Pozzer Krumenauer*

Concerning the paper Laryngomalacia surgery: a series from a tertiary pediatric hospital, issue 78 (6), page 99, the correct name of the author is José Faibes Lubianca Neto.

